# Question and Answer: An Anniversary Interview with Jane Gitschier

**DOI:** 10.1371/journal.pgen.1001018

**Published:** 2010-07-22

**Authors:** Jane Gitschier

**Affiliations:** Department of Medicine and Pediatrics, University of California San Francisco, San Francisco, California, United States of America

## Introduction by Gregory P. Copenhaver

*Important interviews sometimes become iconic—think of Frost interviewing Nixon. More rarely, the interviewers themselves become ingrained in the cultural landscape—think of Mike Wallace from 60 Minutes or Terry Gross from NPR. Here at* PLoS Genetics, *Jane Gitschier (* *Image 1* *) has been conducting published interviews for five years, and her voice has become part and parcel with the journal. To mark the five-year anniversary of the journal's launch, and of Jane's interviews, she has turned the tables and interviewed herself to allow our readership to get to know her and the process a little bit better.*

**Figure:**
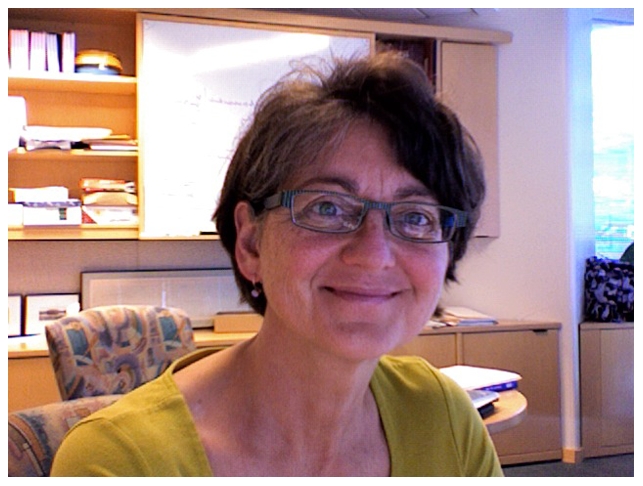
Jane Gitschier.

**Gitschier:** How did you get involved in this interviewing business?

**Gitschier:** Well, I am an inveterate record keeper. I'm the kind of person who actually keeps a table of contents for her lab notebooks and who writes the date on every page. It's genetic—I get it from my dad. He used to keep numerical records of every photo he ever took and a daily log of the stock reports, and he had kept his pay stubs back to when he was a young man in the early 1940s. He got me started on keeping a diary when I was only five years old, and I still keep a journal. So I have this thing about preservation of records.

A couple of things kind of converged. A few years ago, my long-term HHMI funding was about to disappear, which meant that my lab was being forced to shrink considerably. So I started looking for some way to spend some of my energy that wouldn't require a ton of grant money. I applied to be the editor of the *American Journal for Human Genetics*, and one of the things I really wanted to do for the *Journal* was to move into more historical pieces, much in the way *Genetics* has now done. I felt sure I was going to get the position, but I didn't.

Meanwhile, I was invited to introduce Lou Kunkel for the Allan Award at the annual meeting of the American Society of Human Genetics in Toronto in 2004. It was exactly 20 years after I had first seen Lou give a talk on trying to clone the Duchenne muscular dystrophy gene at the first ASHG meeting I had attended—we were speakers in the same session, also in Toronto. I was, and continue to be, very impressed with Lou, so I decided to fly to Boston and interview him in preparation for the introduction.

It was a really moving experience for me, and I think for Lou, too. It is so rare to take the time to simply listen to someone's life story uninterrupted for one or two hours. I read Lou's old papers, took some photographs, did some research. All that for a seven-minute introduction.

My friend Mark Patterson, who is the Director of Publishing for the PLoS family of journals, happened to be in the audience, and afterwards he asked me whether I would be interested in writing interviews for *PLoS Genetics*, a new journal they were about to launch the following summer. So, here was someone who was asking me to do exactly what I wanted to do. And not only that, Mark seemed to have faith in me!

The thing is, I just love knowing about people. And scientists are some of the most interesting people out there. For the most part, they are honest, inquisitive, and obviously smart. It's pretty much “what you see is what you get.” I like that. And the lives they lead—how challenging, how stimulating! And the discoveries that are being made—how astonishing! I wanted to be able to capture some of that.

I also knew that scientists generally don't take the time to write about their experiences. Quite a few years ago now, I was asked by a hematology journal [*Journal of Haemostasis and Thrombosis*] to write about the work I had done on hemophilia while I was a post-doc, a wonderful and productive period of my life, and I really enjoyed taking the time to jumpstart my memory, to talk to other people involved in the discoveries and pick their brains, and to synthesize all this into a cohesive pair of articles. I thought that if I could facilitate this experience for others, it would be a real opportunity to preserve some of their thinking about discovery in an informal setting that is complementary to the actual scientific publication record.

Anyway, that's a pretty long answer to a short question.

**Gitschier:** So how do you choose whom to interview?

**Gitschier:** Well, I get asked that all the time. It's not a very scientific process. I have looked for a well-rounded portfolio, if you will—a collection of people who work on a wide variety of topics, people at a variety of institutions, scientists who have gone on to secondary careers outside of the lab, the president of a university, or an author. And then, a handful of other people whose work somehow dovetails with genetics—a journalist, an historian of science, a judge. Pretty much people that I think will have something interesting to say.

Initially, I asked only people whom I already knew, because that just felt safer. But once I realized how easy it was for me to engage with someone, I got some wind in my sails and I just started asking all kinds of people! What was really amazing was that every single person I asked, with only one exception, said, “Yes!” How cool is that? Of course, ultimately I wasn't able to interview every single one who had said “yes”. In a few cases, we could just never find a suitable time to meet, and in a few other cases, the person just dropped off the radar screen and stopped answering my emails. And one time, unfortunately, after the interview was written up and ready to go, the interviewee decided against publishing it. That was so discouraging; it really set me back for a while.

**Gitschier:** What is the interview process you use?

**Gitschier:** When I invite someone for an interview, I tell him or her up front what is going to happen. I always try to do the interview in person, preferably on his or her home turf, and this has worked out with only a few exceptions. I tape the full interview, except sometimes the interviewee asks me to turn off the recorder when we get to a really juicy bit. Then when I get back to my office, I transcribe the tape. Typically, I have a digital file, and I use two Macs—one serves as the audio player and the other has the word document, and I use my little left finger to start and stop the tape using the space bar. I typically transcribe about 90% of the interview, not bothering to transcribe parts I know I'll never use. You know, every hour of tape is quite a few hours of transcription. I could farm it out, I suppose, but there is no budget for that, and the truth is, I really enjoy reliving the interview and thinking about how the finished product will emerge while I'm transcribing—what to keep, what to shed.

That's one of the fun parts—taking this sometimes meandering mess, which is usually about 7,000 to 10,000 words, and getting it trimmed down and reorganized into some kind of cohesive entity of 3,500–4,000 words. Then I come up with a title and a few paragraphs of introduction and send it back out to the interviewee for his or her clarification, verification, suggestions, etc. I ask people to try to tread lightly on the document, because it is conversational, after all, and most people come back with only some minor suggestions. But sometimes I get a *lot* of red ink. Then it's a lot of back and forth and very time consuming.

But I want people to have the opportunity to be clear and to like the product. After all, it is a collaboration. The *PLoS Genetics* editors also weigh in on it, often helping me to identify places where the interview lags and can be trimmed, and I'm so thankful for that. Also, I always try to remember to take a picture of my subject. Most people are pretty good about that, even though we all know we don't like to have our pictures taken.

**Gitschier:** So what you are saying is that it is just like RNA processing. There is a primary transcript, then a lot of splicing, and some editing, and even some capping.

**Gitschier:** Yes, how clever you are!

**Gitschier:** What kind of audience do you feel you are writing for?

**Gitschier:** Well, obviously, these interviews are part of the “front matter” for a genetics journal, so I know that minimally I'm writing for people who are geneticists. This is also the reason that I wanted to bring in people who were non-geneticists, so that we, as geneticists, can widen our perspective. But since everything with PLoS is freely available online, I also want to be sure to define the jargon and be as clear as possible. I'm hoping that these interviews might be inspirational for students in the field and also of interest to the layperson, if they happen to bump into it online.

**Gitschier:** Are there any kind of themes that have emerged from the interviews with scientists?

**Gitschier:** Yes, there are several things that have really struck me. The first is how often people said that they were doing a project in secret as a graduate student or post-doc. This doesn't always come through in the finished interview, but certainly Svante Pääbo and Adrian Bird are a few examples. The other thing is how often people discover something accidentally—a byproduct of what they are really after. Tom Cech discovering RNA catalysis, Victor Ambros finding microRNA, Herb Boyer bumping into restriction and modification. A lot of big discoveries are made by people willing to think about the data that just don't conform to the expectation. And the third is the profound influence of a high school teacher. This is really a recurring theme; I see it not only in the interviews I've done, but also in the ones I've read or listened to online. Again, it doesn't always show up in the finished product, but it is certainly in evidence. And to all those teachers, I say, “Hallelujah!” I get choked up whenever I hear the story of an inspirational teacher—that is really a life worth living. It has made me think about teaching high school after I retire from doing science.

**Gitschier:** I notice your daughter Annie figures in your interviews occasionally.

**Gitschier:** Yes, Annie is a very important part of my life, of course. Annie's father, my late husband Roy Steinberg, died when she was only three and a half. So, single parenthood has actually put a lot of constraints on the interviews, since I've wanted to do them face-to-face. My travel is very limited, so I'm always thinking ahead to a location where I will happen to be, to see who is out there that I can rope into an interview. I did four interviews as spokes from visiting my father in Pennsylvania and two when visiting my sister in Wisconsin, for example.

So, Annie gets kind of dragged around with me. She's a great sport and exceedingly curious herself, so it has been fun for both of us. And she picks up on these people; she'll say, “Oh he's the serious one,” or “He's the messy one.”

**Gitschier:** OK, last question. Do you have a favorite interview?

**Gitschier:** Well, I get asked that all the time, too. But Jane, you know I can't answer that! The truth is that each person's story is remarkable in its own way. I can hear all of their voices. I am so grateful to these people who put their trust in me. This project has been one of the most rewarding experiences of my life.

